# Opportunities for direct historical delabeling of penicillin allergy in hospitalized patients: an analysis of a U.S. veteran cohort

**DOI:** 10.1017/ash.2026.10829

**Published:** 2026-09-01

**Authors:** Reuben J. Arasaratnam, Christine Vu, Carlos A. Alvarez

**Affiliations:** 1 UT Southwestern: The University of Texas Southwestern Medical Center, USA; 2 Texas Tech University Health Sciences Center Jerry H Hodge School of Pharmacy, USA; 3 Center of Excellence in Real-World Evidence, Texas Tech University Health Sciences Center, Dallas, TX, USA

## Abstract

In over 380,000 U.S. veterans from 2014 to 2024 with a listed penicillin allergy, we report the characteristics and potential antimicrobial stewardship implications of 2781 veterans that could have undergone direct removal of the penicillin allergy label based on the receipt of a penicillin-class antibiotic since the index allergy listing.

## Introduction

Given the adverse consequences of a penicillin allergy label, unverified penicillin allergy labels should be evaluated and removed from a patient’s medical record where feasible.^1^ In the Veterans Health Administration (VHA), several penicillin allergy delabeling programs have been initiated^2^ but insufficient time and resources are implementation barriers particularly when penicillin skin testing or direct oral amoxicillin challenge is required.^3–5^ Direct historical delabeling of a penicillin allergy is defined as the removal of a penicillin allergy label based on history and medical record review alone without the need for penicillin allergy testing. Patients who report non-immune related side effects of penicillin administration, a family history of penicillin allergy, or have proven subsequent safe tolerance of the implicated penicillin since the index allergic reaction are eligible for this delabeling approach.^6^

For Veterans Affairs (VA) medical facilities initiating penicillin allergy evaluation initiatives, direct delabeling is a high-yield approach with minimal barriers to implementation. However, few data exist to inform the opportunity and potential impact of such a delabeling approach within this health system. Thus, we sought to describe the direct historical delabeling opportunities amongst U.S veterans with a listed penicillin allergy and secondarily explore the potential antimicrobial stewardship impact of this delabeling approach by quantifying the ongoing use of high-risk antimicrobials for *Clostridioides difficile* in these individuals.

## Methods

### Study design and definition of cohort

Using patient data from the VA Corporate Data Warehouse, we constructed a retrospective cohort over 2014–2024 of U.S Veterans (≥ 18 yr of age) with a listed penicillin allergy and actively engaged in VA care using previously published definitions.^7^ We utilized the Bar Code Medication Administration (BCMA) log to identify those who had received one or more doses of (a) oral amoxicillin, amoxicillin-clavulanate, penicillin V potassium or oral dicloxacillin (b) intramuscular benzathine penicillin G or (c) intravenous ampicillin, ampicillin-sulbactam, oxacillin and nafcillin, Penicillin GK or piperacillin-tazobactam as an inpatient since the original penicillin allergy listing. To minimize the possibility that these inpatient administrations of penicillin-class antibiotics were part of a formal penicillin allergy delabeling process, we excluded U.S. veterans undergoing oral penicillin challenge^7^ and those with history of Current Procedural Terminology (CPT) codes used in relation to penicillin skin testing or oral amoxicillin challenge (CPT 95018, 95004 and 95076). To ensure the cohort reflected patients with minimal drug allergy module modification, we excluded those with any preexisting comments in the drug allergy module indicating tolerance to penicillin-class antibiotics and those with any modification of the drug allergy listing following the penicillin administration. A flow diagram to indicate the selection of our final cohort is shown in Supplemental Figure 1.

### Outcomes

We obtained data on demographic characteristics, comorbidities, details of the listed penicillin allergy and classification of penicillin allergy reaction using a text mining analysis of the drug allergy module.^7^ To explore evidence of suspected anaphylactic reaction to the penicillin-class antibiotic administered since the allergy listing, we searched the BCMA log for administration of intramuscular epinephrine or lab testing for serum tryptase within 24 hours after administration of the penicillin. We also collected data on antibiotic therapy that has previously been reported as high risk for *Clostridioides difficile* ^8^ (lincosamides [clindamycin], quinolones, third and fourth generation cephalosporins and carbapenems) administered in the three years following the penicillin. Patient characteristics were reported using mean (with standard deviation), medians (with interquartile range IQR) for continuous variables, and frequency and percentage for categorical variables. The study was approved by the VA North Texas Health Care System Institutional Review Board and Research and Development Committee. A waiver of consent was granted.

## Results

Out of a total cohort of 380,160 U.S. veterans with a listed penicillin allergy, we identified 2,781 who were admitted as an inpatient and received at least a single dose of a penicillin-class antibiotic without label modification (Supplementary Figure 1). The characteristics of this cohort are shown in Table 1. The majority were male (n = 2,520, 91%), White (n = 2010, 72%), with an average (mean) age of 62 years and mean Charlson Comorbidity Index of 1.8 (Supplementary Table 1). Penicillin (n = 2,080, 75%) and amoxicillin (n = 417, 15%) were the most frequently listed penicillin-class antibiotics listed in the drug allergy module with most of these entries based on historical patient self-report.

**Table 1.** Characteristics of patients with listed penicillin allergy between 2014 and 2024 who subsequently received one or more penicillin-class antibiotics as an inpatient (n = 2,781) Table 1 long description.

Characteristic	n = 2,781
Age, years, mean (SD)	62 (12.6)
Sex, no. (%)	
Male	2,520 (90.6)
Female	261 (9.4)
Race, no. (%)	
Black or African American	527 (19.0)
Other^a^	75 (2.7)
Missing/unknown	169 (6.1)
White	2010 (72.3)
Ethnicity, no. (%)	
Hispanic or Latino	110 (4.0)
Not Hispanic or Latino	2,552 (91.8)
Other^b^	119 (4.3)
Charlson comorbidity index, mean (SD)	1.8 (2.0)
Type of penicillin-class antibiotic allergy documented in EHR^c^	
Ampicillin (including ampicillin-sulbactam)	82 (2.9)
Amoxicillin (including amoxicillin-clavulanate)	417 (15.0)
Other^c^	115 (4.1)
Penicillin	2,080 (74.8)
Piperacillin (including piperacillin-tazobactam)	88 (3.2)
Average duration (years) of penicillin allergy listing at the time of penicillin administration (mean, SD)	7.5 (7.0)
Documented historical no. (%)/observed penicillin allergy No. (%)	2,748 (98.8) / 33 (1.2)
Presence of any comment regarding allergic reaction no. (%)^d^	
Anaphylaxis	12 (0.9)
Angioedema	8 (0.6)
Cutaneous manifestation	219 (16.2)
Gastrointestinal manifestation	27 (2.0)
Oropharynx and/or throat symptom manifestation	29 (2.2)
Other reaction (neurological manifestation, swelling, or unknown)	127 (9.4)
Respiratory manifestation	21 (1.6)
Concurrent cephalosporin allergy listing in EHR	127 (4.6%)
Concurrent sulfonamide allergy listing in EHR	36 (1.3%)

a Other categories: American Indian, Alaskan Native, Asian, Native Hawaiian, Other Pacific Island or Multiracial.

b Other categories: Unknown, Missing, Declined to Answer.

c Other penicillin-class antibiotics listed were Ticarcillin, Carbenicillin, Methicillin, Nafcillin, Oxacillin and Dicloxacillin. One patient had a listed allergy to both amoxicillin and dicloxacillin.

d 1,431 out of 2,781 did not have any reaction classification documented resulting in a denominator of 1,350 from which these reactions classifications are reported. The reaction categories reported are not mutually exclusive as patients may report more than one reaction to a penicillin-class antibiotic.

EHR, electronic health record; SD, standard deviation.

The penicillin most frequently administered as an inpatient after the index penicillin allergy listing was amoxicillin-clavulanate (n = 1923, 69%), followed by amoxicillin (n = 606, 21.8%) at an average (mean) of 7.5 years since the original listing of the penicillin allergy (Table 2). Notably, there were no doses of intramuscular epinephrine administration recorded in the BCMA log or serum tryptase testing ordered or received within 24 hours of this penicillin administration. Among this cohort with a listed penicillin allergy subsequently receiving a penicillin-class antibiotic, 1,265 patients (45.5%) received antibiotics associated with development of *Clostridioides difficile* during subsequent inpatient admissions over the following 3 years (Supplementary Table 2) including clindamycin (n = 263, 20.8%) and fluoroquinolones (n = 652, 51.5%).

**Table 2.** Subsequent penicillin-class antibiotic administered after original penicillin allergy listing (n = 2,781) Table 2 long description.

Inpatient penicillin administered^a^	n = 2,781^a^	No. of doses, median (IQR)
**Oral**		
Amoxicillin	606 (21.8%)	4 (1–13)
Amoxicillin-clavulanate	1923 (69.2%)	4 (2–9)
Dicloxacillin	6 (0.2%)	1 (1–1)
Penicillin V potassium	48 (1.7%)	12.5 (3–26)
**Intramuscular**		
Penicillin G benzathine	3 (0.1%)	1 (1–2)
**Intravenous**		
Ampicillin	12 (0.4%)	7 (2–28)
Ampicillin-sulbactam	67 (2.4%)	8 (3–15)
Oxacillin and Nafcillin	7 (0.3%)	18 (9–26)
Penicillin GK	63 (2.3%)	15 (6–70)
Piperacillin-tazobactam	239 (8.6%)	11 (4–21)

a 2,596 patients received one penicillin-class antibiotic, 177 patients received 2 different penicillin-class antibiotics, and 8 patients received 3 different penicillin-class antibiotics, resulting in a total of 2,974 distinct penicillin-class antibiotic courses.

## Discussion

In our study, we identified 2,781 potential candidates for direct delabeling of their penicillin allergy based on subsequent inpatient administration of penicillin since their index penicillin allergy listing without evidence of label modification. 46% of these patients later received antibiotics associated with higher risk of *Clostridioides difficile*, possibly representing a lingering consequence of a persistent unverified penicillin allergy label. Taken together, ample opportunity exists within the VHA for direct delabeling based on medication administration history alone with potential to improve antimicrobial stewardship within this population.

Direct delabeling has been shown to be an effective approach in several studies. In a multicenter penicillin allergy delabeling initiative from Australia during 2019, 161 of 355 (45%) patients were able to have their penicillin allergy removed directly without the need for formal testing.^9^ Within the VHA, several individual facilities have also used this as part of their penicillin allergy evaluation process, facilitating the immediate use of penicillin-class antibiotics.^5,10^ This approach to delabeling may be particularly suited to VA facilities given the longstanding interconnected electronic health record of the health system, enabling providers to prove previous safe tolerance of penicillin-class antibiotics up to several decades ago.^2^ Our findings suggest this approach could be further optimized by preferentially focusing on those that have tolerated amoxicillin or amoxicillin-clavulanate since their penicillin allergy listing.

We acknowledge the limitations of this report. We intentionally restricted our analysis to inpatient penicillin administration which could be verified via the BCMA log. Further opportunities for direct penicillin allergy delabeling likely exist in the outpatient setting. While we included patients who had received piperacillin as potential targets for direct penicillin allergy delabeling, these patients may warrant further testing prior to label removal as cross-tolerance between piperacillin and penicillin is less well established than between the aminopenicillins and penicillin. As individual chart review was not performed, this limits the ability to explore reasons behind antimicrobial prescribing and to detect potential adverse reactions to the administered penicillin outside of our analysis (such as epinephrine administration outside the BCMA log). Finally, this study does not capture additional direct penicillin allergy delabeling opportunities from non-VA facility healthcare encounters.

In summary, ample opportunities exist for direct penicillin allergy delabeling within the VHA which may be a feasible, high-yield penicillin allergy evaluation approach, with potential positive impact on antimicrobial use.

## Supporting information

10.1017/ash.2026.10829.sm001 Arasaratnam et al. supplementary material 1 Arasaratnam et al. supplementary material

10.1017/ash.2026.10829.sm002 Arasaratnam et al. supplementary material 2 Arasaratnam et al. supplementary material

10.1017/ash.2026.10829.sm003 Arasaratnam et al. supplementary material 3 Arasaratnam et al. supplementary material

## Data Availability

The data used in this study are maintained within the U.S. Department of VA secure data environment and are not publicly available. Access to VA data is restricted to authorized investigators with an approved VA research protocol and appropriate data use agreements.

## Supplementary material

The supplementary material for this article can be found at https://doi.org/10.1017/ash.2026.10829.

## Table 1. Long description

The demographic and clinical characteristics are shown of 2781 patients with a listed penicillin allergy that received 1 or more penicillin class antibiotics since the index allergy listing. The mean age of this cohort was 62 years (standard deviation 12.6). The cohort was predominantly male patients (n=2520) with a mean Charlson Comorbidity Index of 1.8 (standard deviation 2.0). Penicillin was the most frequently listed class of penicillin allergy documented in the electronic health record (n=2080). The mean duration of years of penicillin allergy listing at the time of subsequent penicillin administration was 7.5 years (standard deviation 7.0).

Navigate back to Table 1..

## Table 2. Long description

Our of 2781 patients that had received a penicillin class antibiotic since the original listing, this table shows that amoxicillin-clavulanate was the most frequently administered in 1923 patients with a median dose number of 4 (interquartile range 2-9) followed by amoxicillin administered in 606 patients with a median dose number of 4 (range 1-13)

Navigate back to Table 2..

## References

[1] Khan DA , Banerji A , Blumenthal KG , et al. Drug allergy: a 2022 practice parameter update. J Allergy Clin Immunol 2022;150:1333–1393. 10.1016/j.jaci.2022.08.028 36122788

[2] Kouma MA , Guastadisegni JM , Yang L , Maxwell DN , Storey DF , Arasaratnam RJ. Challenges and opportunities related to penicillin allergy in the Veterans Health Administration: a narrative review. Antimicrob Steward Healthc Epidemiol 2023;3:e174. 10.1017/ash.2023.448 PMC10644167 38028897

[3] Gillespie C , Sitter K , McConeghy KW , et al. Facilitators and barriers to verifying penicillin allergies in a veteran nursing home population. J Allergy Clin Immunol Pract 2023;11:2848–2854 e2843. 10.1016/j.jaip.2023.06.023 37352930

[4] Alagoz E , Saucke M , Balasubramanian P , Lata P , Liebenstein T , Kakumanu S. Barriers to penicillin allergy de-labeling in the inpatient and outpatient settings: a qualitative study. Allergy Asthma Clin Immunol 2023;19:88. 10.1186/s13223-023-00842-y PMC10568923 37821953

[5] Arasaratnam RJ , Guastadisegni JM , Kouma MA , Maxwell D , Yang L , Storey DF. Rising to the challenge: an ID provider-led initiative to address penicillin allergy labels at a large veterans affairs medical center. Open Forum Infect Dis 2024;11:ofae396. 10.1093/ofid/ofae396 PMC11310584 39130085

[6] Joean O , Sermet K , Ashkenazi-Hoffnung L , et al. ESCMID clinical guidelines on the evaluation and management of a reported antibiotic allergy. Clin Microbiol Infect 2026;32:767–787. 10.1016/j.cmi.2026.02.011 41707748

[7] Arasaratnam RJ , Vu C , Alvarez CA. Temporal trends of inpatient oral penicillin challenges in a U.S. veteran cohort with a recorded penicillin allergy. Antimicrob Steward Healthc Epidemiol 2025;5:e297. 10.1017/ash.2025.10205 PMC12616559 41245843

[8] Tabak YP , Srinivasan A , Yu KC , et al. Hospital-level high-risk antibiotic use in relation to hospital-associated Clostridioides difficile infections: retrospective analysis of 2016-2017 data from US hospitals. Infect Control Hosp Epidemiol 2019;40:1229–1235. 10.1017/ice.2019.236 PMC9390868 31522695

[9] Chua KYL , Vogrin S , Bury S , et al. The penicillin allergy delabeling program: a multicenter whole-of-hospital health services intervention and comparative effectiveness study. Clin Infect Dis 2021;73:487–496. 10.1093/cid/ciaa653 PMC8326579 32756983

[10] Mitchell AB , Ness RA , Bennett JG , et al. Implementation and impact of a beta-lactam allergy assessment protocol in a veteran population. Fed Pract 2021;38:420–425. 10.12788/fp.0172 PMC8562903 34737539

