# Supplementary material for: Opportunities for direct historical delabeling of penicillin allergy in hospitalized patients: an analysis of a U.S. veteran cohort
Source: Antimicrob Steward Healthc Epidemiol. 2026 Sep 1;6(1):e250. doi: 10.1017/ash.2026.10829 (PMC13760384; doi:10.1017/ash.2026.10829)
Supplement: Supplementary file 1 [file S2732494X26108298sup002.docx]

**Supplementary Table 1. Charlson Comorbidity Index Components of the Cohort (n=2781)**

| **Comorbidity^a^** | **No. (%)** |
| --- | --- |
| AIDS/HIV | 41 (1.5) |
| Any malignancy | 276 (9.9) |
| Cerebrovascular disease | 235 (8.5) |
| Chronic pulmonary disease | 697 (25.1) |
| Congestive heart failure | 282 (10.1) |
| Dementia | 20 (0.7) |
| Diabetes (with chronic complications) | 354 (12.7) |
| Diabetes (without chronic complications) | 1033 (37.1) |
| Hemiplegia/Paraplegia | 109 (3.9) |
| Metastatic Solid Tumor | 13 (0.5) |
| Mild liver disease | 177 (6.4) |
| Moderate or Severe Liver disease | 18 (0.6) |
| Myocardial infarction | 96 (3.5) |
| Peptic ulcer disease | 29 (1.0) |
| Peripheral vascular disease | 273 (9.8) |
| Renal disease | 282 (10.1) |

^a^Categories are not mutually exclusive; percentages add to more than 100%

Abbreviations: AIDS, Acquired Immunodeficiency Syndrome; HIV, Human Immunodeficiency Virus.
