# Supplementary material for: Opportunities for direct historical delabeling of penicillin allergy in hospitalized patients: an analysis of a U.S. veteran cohort
Source: Antimicrob Steward Healthc Epidemiol. 2026 Sep 1;6(1):e250. doi: 10.1017/ash.2026.10829 (PMC13760384; doi:10.1017/ash.2026.10829)
Supplement: Supplementary file 2 [file S2732494X26108298sup003.docx]

**Supplementary Table 2. Inpatient high-risk antibiotic administration in the following three years after penicillin administration (n=1265)^a^**

| **Antibiotics associated with higher risk for *Clostridioides difficile* administered in following 3 years** | **N = 1265^b^** | **No. of Doses, Median (IQR)** |
| --- | --- | --- |
| Lincosamides | 263 (20.8%) | 8 (3-20) |
| Fluoroquinolones | 652 (51.5%) | 5 (2-12) |
| Third generation cephalosporins | 611 (48.3%) | 4 (2-8) |
| Fourth generation cephalosporins | 388 (30.7%) | 6.5 (3-15) |
| Carbapenems | 209 (16.5%) | 13 (5-26) |

^a^556 patients out of the total cohort of 2781 received a penicillin in the calendar years 2022-2024 and had insufficient follow-up time to complete the planned 3-year observation period for high-risk antibiotic administration.

^b^2,123 courses of distinct classes of higher risk antibiotics were given to 1265 patients: 675 patients received one class of higher risk antibiotics, 379 patients received two classes, 160 patients received three classes, 45 received four classes, 6 patients received five classes of higher risk antibiotics.
